# Chemogenetic stimulation of phrenic motor output and diaphragm activity

**DOI:** 10.1101/2024.04.12.589188

**Published:** 2024-04-15

**Authors:** Ethan S. Benevides, Prajwal P. Thakre, Sabhya Rana, Michael D. Sunshine, Victoria N. Jensen, Karim Oweiss, David D. Fuller

**Affiliations:** 1 Department of Physical Therapy, University of Florida, Gainesville, FL, 32601; 2 Breathing Research and Therapeutics Center, University of Florida, Gainesville, FL, 32601; 3 McKnight Brain Institute, University of Florida, Gainesville, FL, 32601; 4 Department of Electrical and Computer Engineering, University of Florida, Gainesville, FL, 32601

**Keywords:** DREADD, Chemogenetics, Phrenic, Diaphragm EMG, Plethysmography, Adeno-associated virus, Respiratory neural control

## Abstract

Impaired diaphragm activation contributes to morbidity and mortality in many neurodegenerative diseases and neurologic injuries. We conducted experiments to determine if expression of an excitatory DREADD (designer receptors exclusively activation by designer drugs) in the mid-cervical spinal cord would enable respiratory-related activation of phrenic motoneurons to increase diaphragm activation. Wild type (C57/bl6) and ChAT-Cre mice received bilateral intraspinal (C4) injections of an adeno-associated virus (AAV) encoding the hM3D(Gq) excitatory DREADD. In wild type mice, this produced non-specific DREADD expression throughout the mid-cervical ventral horn. In ChAT-Cre mice, a Cre-dependent viral construct was used to drive DREADD expression in C4 ventral horn motoneurons, targeting the phrenic motoneuron pool. Diaphragm EMG was recorded during spontaneous breathing at 6–8 weeks post-AAV delivery. The selective DREADD ligand JHU37160 (J60) caused a bilateral, sustained (>1 hr) increase in inspiratory EMG bursting in both groups; the relative increase was greater in ChAT-Cre mice. Additional experiments in a ChAT-Cre rat model were conducted to determine if spinal DREADD activation could increase inspiratory tidal volume (VT) during spontaneous breathing without anesthesia. Three to four months after intraspinal (C4) injection of AAV driving Cre-dependent hM3D(Gq) expression, intravenous J60 resulted in a sustained (>30 min) increase in VT assessed using whole-body plethysmography. Subsequently, direct nerve recordings confirmed that J60 evoked a >50% increase in inspiratory phrenic output. The data show that mid-cervical spinal DREADD expression targeting the phrenic motoneuron pool enables ligand-induced, sustained increases in the neural drive to the diaphragm. Further development of this technology may enable application to clinical conditions associated with impaired diaphragm activation and hypoventilation.

## INTRODUCTION

Many respiratory disorders are associated with reduced or impaired activation of respiratory motoneurons. Neurologic injuries (e.g., traumatic spinal cord injury, stroke) and neurodegenerative conditions (e.g., ALS, Pompe disease) will often result in decreased respiratory motor output, including impaired activation of the phrenic motoneurons which innervate the diaphragm^1–6^. Another prominent example is obstructive sleep apnea, in which pharyngeal motoneurons have reduced output during sleep^7^. Treatment options that target increased respiratory motoneuron activation to improve breathing ability are limited. However, designer receptors exclusively activated by designer drugs (DREADDs) may have use in this regard^8^. Structurally derived from naturally occurring G-protein coupled receptors, DREADDs have been engineered to respond exclusively to exogenous ligands that are otherwise biologically inert^9–11^. This provides a means to selectively stimulate cells expressing the DREADD. Prior studies have used DREADDs to stimulate upper airway muscle activation during breathing^8^. For example, following expression of DREADDs in murine hypoglossal motoneurons, tongue electromyogram (EMG) activity can be increased using DREADD ligands^12–16^. This response is functionally beneficial as shown by increased patency of the upper airway^12^.

The present study focused on chemogenetic activation of the phrenic neuromuscular system. Phrenic motoneurons provide motor innervation of the diaphragm muscle and are located in the mid-cervical (C3–5) spinal cord^17^. We tested the hypothesis that expressing DREADDs in the mid-cervical spinal cord would enable systemic (intravenous) delivery of a selective DREADD ligand to produce sustained increases in the respiratory-related activation of the diaphragm. In doing so, we addressed two important questions. First, we determined if effective diaphragm activation requires focal DREADD expression targeting phrenic motor neurons, or if non-specific expression in the immediate vicinity of the phrenic motor nucleus would be sufficient. This question derives from prior studies of cervical spinal cord stimulation. A compelling body of work, with studies in multiple species, demonstrates that non-specific activation of cervical spinal networks can be highly effective at increasing diaphragm activation^18–20^. One theory to explain this result is that a general increase in excitability of cervical propriospinal networks leads to increased phrenic motoneuron activation^21–23^. There is also evidence that phrenic motoneurons can integrate multiple synaptic inputs in a manner that produces orderly recruitment^18^. Accordingly, DREADD-induced activation of mid-cervical neurons or networks may be sufficient for ligand-induced diaphragm activation. On the other hand, DREADD expression may need to be restricted to phrenic motoneurons if the goal is to produce inspiratory-related diaphragm activation. To address this question, we studied diaphragm responses in two mouse models: 1) a wild type model in which DREADDs were non-specifically expressed in the C3–5 spinal cord, encompassing mid-cervical interneurons and motoneurons, and 2) a choline acetyltransferase (ChAT)-Cre transgenic model in which DREADD expression was restricted to ChAT-positive neurons in the anterior C3–5 spinal cord, targeting the phrenic motoneuron pool.

The second question we addressed was if phrenic motoneuron activation via cervical spinal cord DREADDs could produce a sustained increase in inspiratory tidal volume in unanesthetized, spontaneously breathing animals. While the previous results from the hypoglossal motor system^12,14–16^ provide a proof-of-concept that DREADDs can stimulate respiratory motoneuron activity, whether a *sustained* increases in tidal volume could be evoked by expressing DREADDs in phrenic motoneuron was uncertain. For example, during spontaneous breathing, a DREADD-induced increase in phrenic motoneuron excitability, and thus diaphragm activation, could be rapidly offset by decreases in bulbospinal neural drive to the phrenic motor pool, secondary to reduced arterial CO_2_ or increased vagal-mediated inhibition. An increase in diaphragm activation could also trigger a decrease in accessory respiratory muscle activation, thereby attenuating or preventing increases in tidal volume. Lastly, data from the hypoglossal motor system^12,14–16^, as well as our initial results in the anesthetized mouse indicated that both phasic (i.e., during the inspiratory period) and tonic (i.e., occurring across the respiratory cycle) activation of the diaphragm would increase after DREADD activation, and how this would impact tidal volume was not clear. Accordingly, we studied ChAT-Cre rats using whole body plethysmography and direct measure of phrenic motor output via nerve recordings. The plethysmography studies allowed us to determine if DREADD activation of phrenic motoneurons causes a sustained increased in tidal volume and ventilation during spontaneous breathing in the unanesthetized rat. The nerve recordings were done under anesthesia and enabled direct quantification of the impact of DREADD activation on neural drive of the diaphragm while controlling variables including arterial CO_2_ and lung volume.

Collectively the results of this work demonstrate that mid-cervical spinal DREADD expression enables the J60 ligand to produce a sustained increase in the neural drive to the diaphragm, producing an increase in tidal volume during spontaneous breathing. Further development of this technology may enable application to clinical conditions associated with impaired diaphragm activation and hypoventilation.

## METHODS

### Animals.

Experiments were carried out using C5/bl6, wild type mice (Taconic), ChAT-Cre transgenic mice (B6.129S6-Chattm2(cre)Lowl/J; Jackson Laboratories), and ChAT-Cre transgenic rats (LE-Tg(Chat-Cre)5.1Deis; Rat Resource & Research Center). Animals were singly housed in a controlled environment (12 h light–dark cycle) with food and water *ad libitum*. All experiments were conducted in accordance with the NIH Guidelines Concerning the Care and Use of Laboratory Animals and were approved by the University of Florida Institutional Animal Care and Usage Committee (protocol #202107438).

### Intraspinal injections.

An adeno-associated viral vector (AAV) encoding the gene for the excitatory DREADD, hM3D(Gq) was delivered to the mid-cervical spinal cord. Surgery was performed under aseptic conditions. Mice were anesthetized with isoflurane (induction: 3–4% isoflurane; maintenance: 2–3% isoflurane in 100% O_2_) while rats were anesthetized with a mixture of ketamine (100 mg/kg) and xylazine (10 mg/kg) delivered intraperitoneally. Animals were placed prone on a circulating water heating pad to maintain body temperature. A longitudinal incision was made starting at the base of the skull and extending caudally. The underlying back musculature was opened from the base of the skull to spinal segment C6. Using a micro-curette, the muscle and connective tissue overlying laminae C3 to C5 were removed. A laminectomy of the C4 dorsal lamina exposed the dura mater below. A bilateral durotomy was then performed exposing the spinal cord. A Hamilton syringe (34-gauge needle) held in a Kopf stereotaxic frame was as used to inject 1 μl of AAV9-hSyn-DIO-hM3D(Gq)-mCherry (ChAT-Cre mice and rats; titer: 2.07×10^12^ vg/mL) or AAV9-hSyn-HA-hM3D(Gq)-mCherry (C57/bl6 mice; titer: 2.44×10^13^ vg/mL), bilaterally into the ventral horns at C4. Injections were made 0.5 mm lateral to the spinal midline at a depth of 0.9 mm for mice^24^ and 1 mm lateral to midline at a depth of 1.5 mm for rats^25^.The needle was left to dwell for 5 minutes. Following injections, the overlying muscle and fascia were sutured with absorbable suture, the skin closed, and the animal returned to its home cage. Animals received a post-operative analgesia regiment of subcutaneous buprenorphine (1 mg/kg; slow-release formulation) and carprofen (5 mg/kg) for the first three days after surgery.

### Diaphragm EMG recordings.

Recordings were conducted using wild type (n= 11; n= 7 females) and ChAT-Cre mice (n= 9; n= 6 females; n= 1 excluded from analysis), 6–8 weeks following intraspinal injections of AAV-DREADD. Animals were anesthetized with 2–3% isoflurane in a closed chamber and then placed supine on a closed loop heating pad to maintain rectal temperature at 37 ± 0.5 °C (model 700 TC-1000, CWE Inc.). Mice spontaneously inhaled 2% isoflurane in 100% O_2_ for the duration of the experiment.

A laparotomy was performed and two sets of 50 μm tungsten wires were placed in the mid-costal region of the left and right hemi-diaphragm. The tips of each wire were de-insulated, bent into small hooks, and inserted through the diaphragm approximately 3 mm apart. The recorded EMG signals were amplified (1000x) and filtered (100–1000 Hz) using a differential amplifier (A–M systems model 1700). Signals were digitized at 10 kS/s using a Power 1401 (CED, Cambridge, UK).

Once a stable plane of anesthesia was reached, animals underwent a 10-minute recording to establish baseline diaphragm EMG parameters. Subsequently, animals received injections of vehicle (100 μl of saline delivered intraperitoneally (IP) followed by a 20-minute recording. Animals then received an intraperitoneal injection of the selective DREADD agonist, JHU37160 (J60; 0.1 mg/kg, HB6261, HelloBio), and recordings continued for 90 minutes. At the conclusion of each experiment, animals underwent transcardial perfusion with saline followed by 4% paraformaldehyde. Following perfusion, spinal cords were harvested for histological analysis.

### Whole body plethysmography.

ChAT-Cre rats (n= 9; n= 3 females) were studied using flow-through whole body plethysmography 14–16 weeks after intraspinal delivery of AAV9-hSyn-DIO-hM3D(Gq)-mCherry, as described above. Unanesthetized rats were sealed into the Plexiglas plethysmograph with airflow maintained at 6 L/min for the duration of the recording. The recording protocol consisted of a 40-minute acclimation period (inspired air: 21% O_2_, 79% N_2_), followed by a 7-minute ventilatory challenge (10% O_2_, 7% CO_2_, 83% N_2_) and a 10-minute normoxic recovery period (21% O_2_, 79% N_2_). Subsequently, rats underwent a 20-minute long, pre-vehicle, baseline under normoxic conditions (21% O_2_, 79% N_2_) followed by intravenous infusion of the J60 vehicle (saline). Following vehicle infusion recording continued for 30-minutes followed by a 7-minute ventilatory challenge (10% O_2_, 7% CO_2_, 83% N_2_) and 10-minutes of normoxic breathing (21% O_2_, 79% N_2_). After an additional 20-minute pre-J60 baseline (21% O_2_, 79% N_2_), an intravenous infusion of the J60 ligand was given (0.1 mg/ml) and recordings continued for 30-minute followed by a final ventilatory challenge (10% O_2_, 7% CO_2_, 83% N_2_). The ventilatory challenges were performed to assess the ability to increase breathing.

### Phrenic nerve recordings.

Two-to-eight-weeks following plethysmography recordings, bilateral phrenic nerve recordings were performed. This procedure was done to directly assess the effect of DREADD activation on phrenic motor output under rigorously controlled experimental conditions. Anesthesia was induced by placing the rat in a closed chamber to inhale 3% isoflurane in 100% O_2_. Rats were then moved onto a closed-loop heating pad set to maintain rectal temperature at 37 ± 1°C (model 700 TC-1000, CWE Inc.). Isoflurane anesthesia was maintained using a nose cone. Once a surgical plane of anesthesia was reached as evidenced by loss of corneal reflexes and hindlimb withdrawal, rats were tracheotomized and ventilated (VentElite, model 55–7040; Harvard Apparatus Inc.) with a gas mixture of 50% O_2_, 1% CO_2_, balanced with N_2_. End-tidal CO_2_ was maintained at 45–47 mmHg throughout the surgery and experimental protocol (Capnogard; Novametrix). Ventilator frequency was maintained between 65 and 75 breaths/min, and tidal volume was set at 7 mL/kg^26^. The vagus nerves were transected bilaterally to prevent entrainment of phrenic efferent output with the ventilator.

A tail vein catheter was placed to allow for intravenous infusion of urethane anesthesia, supplementary fluids, and the J60 ligand. Animals were slowly converted from inhaled isoflurane to urethane anesthesia (2.1 g/kg at 6 mL/hr; IV). During this conversion, the depth of anesthesia was consistently monitored by evaluating the pedal withdrawal reflex. Following administration of the full urethane dose, a mixture of 8.4% sodium bicarbonate and lactated Ringer’s was administered (2 mL/h; IV) to maintain acid-base balance. To prevent movements and EMG contamination of the phrenic neurogram pancuronium bromide was administered (3 mg/kg IV, Sigma-Aldrich, St Louis) to achieve neuromuscular blockade. A catheter (polyethylene tubing; PE 50; Intramedic) was placed in the femoral artery to enable monitoring of arterial blood pressure via a transducer amplifier (TA-100, CWE) and allow withdrawal of arterial blood samples (65 μL) for measurement of partial pressure of CO_2_ (PaCO_2_ ) and O_2_ (PaO_2_ ), pH, and base excess (ABL 90 Flex, Radiometer; Copenhagen, Denmark).

The phrenic nerves were exposed bilaterally using a dorsal approach as described previously^27,28^. Briefly, a midline incision was made at the base of the skull extending to spinal level T2. The muscles connecting the shoulder blades to the spinal column were separated to expose the phrenic nerves. The phrenic nerves were isolated, cut distal to the spinal cord and suctioned into a custom-made glass electrodes filled with 0.9% saline solution. Phrenic nerve activity was amplified (10 kHz) using a differential AC amplifier (Model 1700, A-M systems, Everett, WA), band-pass filtered (100Hz-3 kHz), and digitized at 25ks/second (Power 1401, CED).

At the beginning of the experiment, the apneic threshold was determined by slowly reducing the inspired CO_2_ until phrenic nerve inspiratory activity ceased for 60 seconds. The recruitment threshold was established by slowly increasing the inspired CO_2_ until phrenic bursting returned. The end tidal CO_2_ (ETCO_2_) was then maintained 2–3 mmHg above the recruitment threshold for the duration of the experiment. After achieving a stable phrenic nerve recording and blood gases a 15-minute-long baseline recording was collected (50% O_2_, 3% CO_2_) followed by a brief, 5-minute exposure to hypoxia (11.5% O_2_, 3% CO_2_) and 10–15-minute recovery period (50% O_2_, 3% CO_2_). Subsequently, intravenous infusion of vehicle (saline) was given followed by a 15-minute recording period. The J60 ligand (0.1 mg/kg) was then administered intravenously over a 2 minute infusion period followed by a 100-minute recording period.

Arterial blood samples were collected at specific intervals: initially at baseline, during the last minute of each hypoxia episode, 15 minutes post vehicle administration, and subsequently at 20, 40, 60, 80, and 100 minutes post J60 administration. Baseline blood gas values served as references to assess if further arterial samples were isocapnic. To keep end-tidal CO_2_ and PaCO_2_ near baseline (within ± 2.0 mmHg), minor adjustments to inspired CO_2_ and ventilation rate were made as needed. PaO_2_ was kept above 150 mmHg, except during hypoxia; if it dropped below, O_2_ intake was increased by 5%, and a new blood sample was analyzed within 5 minutes.

At the end of the experiment animals were exposed to a second 5-min episode of hypoxia (11.5% O_2_) followed by a brief “maximal” chemoreceptor challenge induced by switching off the mechanical ventilator until the animal exhibited a “gasping-like” phrenic discharge pattern (approximately 20–30 seconds). If the increase in phrenic nerve amplitude in response to the “maximal” challenge was lower than the response observed during either hypoxic episodes, it was considered a sign of deteriorating nerve-electrode contact and the preparation was excluded from all formal analysis. Rats were then perfused transcardially with heparinized saline followed by 4% paraformaldehyde and spinal cords were removed harvested for histological analysis.

### Histology.

Spinal cords were harvested and placed in 4% paraformaldehyde for 24 hours. The cords were subsequently moved to a cryo-protecting solution (30% sucrose in 1x PBS) for a minimum of three days. Cervical and thoracic spinal cords were blocked in optimal cutting temperature media and cryosectioned at 20 μm. The viral constructs included a red fluorescent protein (mCherry) fused to the hM3D(Gq) DREADD which allowed evaluation of DREADD expression by assessing mCherry expression via fluorescence microscopy.

We performed a qualitative assessment of mCherry expression in the mid-cervical spinal cord. One intact section from the middle of each spinal segment (C3-C6) was chosen as a representative section and underwent assessment. Sections were segmented into the following quadrants: left dorsal, right dorsal, left ventral, and right ventral. The quadrant was scored as “positive” if mCherry positive neurons or fibers were observed; otherwise, the sub-segment was marked “negative”. Results were compiled into a summary table showing the total positive counts by animal cohort, spinal segment, and quadrant (see Results section; Table 1). Animals that showed no positive mCherry labeling in the C3-C6 cord and no EMG response to J60 were excluded from analysis.

### Data analysis.

Custom MATLAB (MathWorks; Natick, MA) scripts were created, and are available upon request. These scripts were used to analyze diaphragm EMG, phrenic nerve, and plethysmography waveforms. EMG signals were digitally filtered using a second-order, bandpass Butterworth filter (100–1000 Hz) and then rectified and integrated by taking the absolute value of the signal followed by applying a moving median filter (50 ms time constant for mice; 75 ms time constant for rats) and moving average filter (50 ms time constant for mice; 175 ms time constant for rats). The script identified each EMG burst and calculated peak amplitude, minimum amplitude (tonic activity), and area under the curve (AUC) for each burst which was then averaged across animals and compared across experimental conditions. Differences in mortality between wild type and ChAT-Cre mice post-J60 application was assessed using Pearson’s Chi-squared test with Yates’ continuity correction using the chisq.test function in R. In instances where animals did not survive the entire duration of the anesthetized recording, data up until the time point preceding their death was included.

Phrenic nerve signals were digitally filtered using a second-order, bandpass Butterworth filter (100–3 kHz) and then rectified and integrated by taking the absolute value of the signal followed by applying a moving median filter (50 ms time constant) and moving average filter (50 ms time constant). The script calculated the peak phrenic burst amplitude and minimum amplitude for each burst which was then averaged across animals and compared across experimental conditions. Systolic (SP), diastolic (DP), and mean arterial blood pressure (MAP; formula: MAP = DP + 1/3 (SP − DP)) along with instantaneous heart rate were calculated from the arterial pressure trace.

In plethysmography experiments, airflow pressure, chamber temperature, chamber humidity, barometric pressure, and animal’s body temperature were used to calculate respiratory frequency, tidal volume, and ventilation via a custom MATLAB script. Tidal volume was calculated using the Drorbaugh and Fenn equation^29^.

Statistical analyses were performed using SigmaPlot 14 (Systat Software) and R (The R Foundation for Statistical Computing; version 4.3.1). In mouse diaphragm EMG studies, one-way repeated measure analysis of variance (ANOVA) was used to statistically compare diaphragm EMG peak amplitude, area under the curve, tonic activity, and heart rate across time before and after J60 application. Two-sample t-tests were used to compare left and right hemi-diaphragm EMG peak amplitude, area under the curve, tonic activity, and heart rate between ChAT-Cre and wild type mice at the 30-minute post-J60 administration time point. In plethysmography experiments, two-way repeated measures ANOVA was used to compare raw and normalized tidal volume, respiratory frequency, and minute ventilation across time and treatment (saline vs J60). One-way RM ANOVA was used to compare phrenic peak amplitude, systolic and diastolic blood pressures, mean arterial blood pressure, and respiratory rate across time for phrenic nerve recordings. The relationship between time post-AAV injection and average phrenic response to J60 was assessed for ChAT-Cre rats using the cor.test function in R to run a Pearson’s product moment correlation.

In cases of significant main effects, Tukey post-hoc test was used to assess differences between individual time points. For instances where data failed to meet general linear model assumptions (i.e., normality, homogeneity of variances), nonparametric equivalents of the previously mentioned statistical tests were used. Data were considered statistically significant when p≤0.05. The mean data are presented along with standard error of the mean.

## RESULTS

### Diaphragm EMG responses in wild type mice.

**Figure 1.**
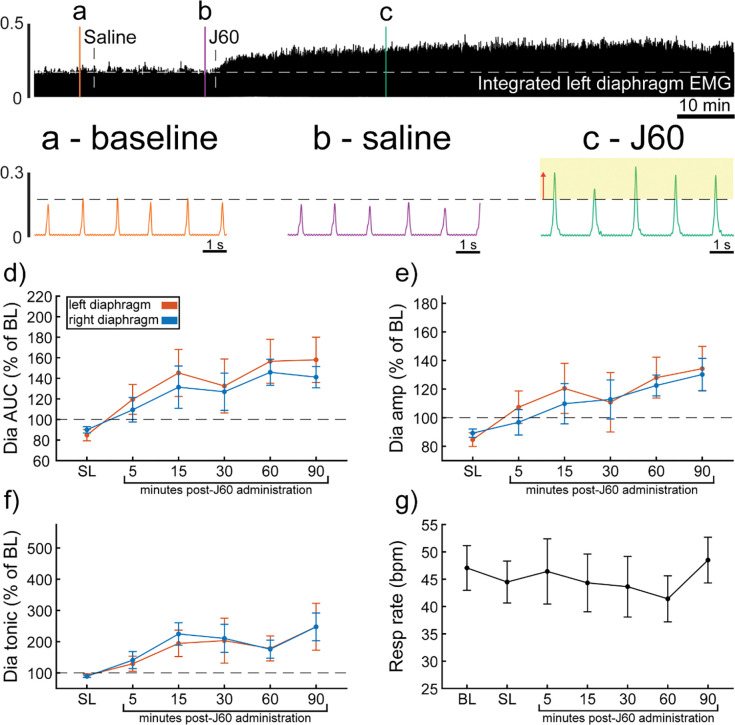
DREADD activation increases diaphragm EMG output in wild type mice. A representative example of diaphragm EMG activity before and after application of the J60 DREADD ligand is shown in the top panel. Examples of the individual inspiratory EMG bursts at baseline (a), after vehicle (b), and after J60 (c) are shown. The J60 ligand increased diaphragm output but did not impact respiratory rate. The mean responses (n= 11; n= 7 females) for EMG AUC, peak-to-peak amplitude, tonic activity, and respiratory rate are shown in panels d-g. For diaphragm EMG data (panels d-f) left hemi-diaphragm EMG is represented in orange, while right hemi-diaphragm EMG is blue. Error bars depict ± 1 SEM. Statistical reports for all panels are provided in Supplemental Table 1. Dia = diaphragm, AUC = area under the curve, amp = peak amplitude, BL = baseline, SL = saline (sham injection).

Wild type mice underwent bilateral injections of AAV9-hSyn-HA-hM3D(Gq)-mCherry into the ventral horns at spinal segment C4. Following a six-to-eight-weeks incubation period, mice underwent terminal diaphragm EMG recordings before and after application of the selective DREADD ligand, J60. On average, wild type mice showed increases in diaphragm EMG output in at least one hemi-diaphragm after J60 administration (Figure 1). Diaphragm EMG AUC was significantly increased after DREADD activation (Figure 1d) in both the left (**p = 0.002**; Table S1) and right (**p = 0.002**; Table S1) hemi-diaphragm. Additionally, peak-to-peak amplitude of the rectified and integrated diaphragm EMG burst activity increased bilaterally following J60 administration (Left hemi-diaphragm: p = 0.056; Right hemi-diaphragm: **p = 0.01**; Figure 1e; Table S1). Lastly, the tonic activity of the diaphragm was assessed (Figure 1f). Similar to the previous measure of EMG output both the left (**p = 0.052**; Table S1) and right (**p < 0.001**; Table S1) hemi-diaphragm exhibited a significant increase in tonic activity following J60 administration. Respiratory rate was consistent for the duration of the experiment. Notably, there was no substantial change in respiratory rate of these spontaneous breathing mice after J60 administration (p = 0.863; Figure 1g; Table S1).

In all experiments, the selective DREADD ligand, J60, produced an increase in diaphragm EMG burst amplitude during inspiration. However, this increase was not always detected in both the left and right hemi-diaphragm EMG recording. Five mice showed a bilateral increase in diaphragm output after J60 administration (Figure 1). Four mice showed a response that was limited to the right hemi-diaphragm while two showed a response that was limited to the left hemi-diaphragm (Figure 1).

### Diaphragm EMG responses in ChAT-Cre mice.

**Figure 2.**
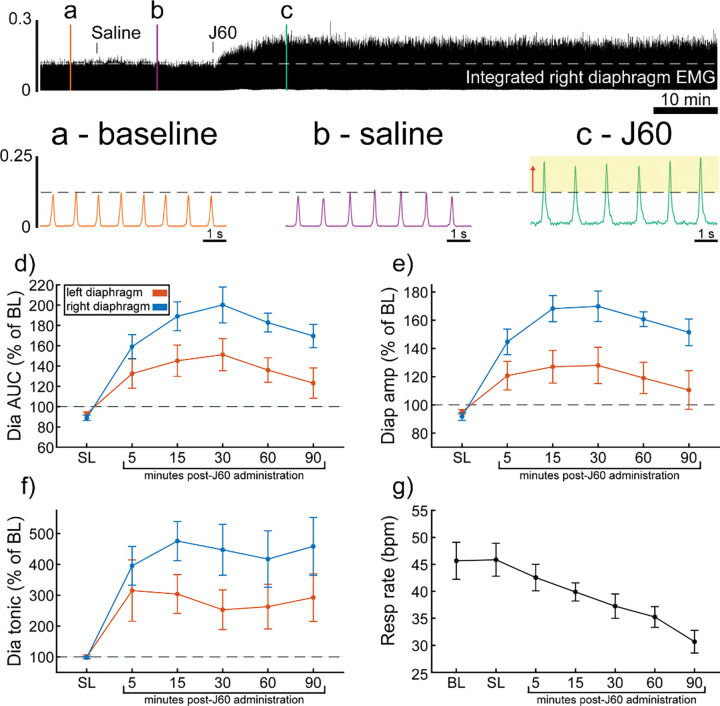
DREADD activation increases diaphragm EMG output in ChAT-Cre mice. A representative example of diaphragm EMG activity before and after application of the J60 DREADD ligand is shown in the top panel. Examples of the individual inspiratory EMG bursts at baseline (a), after vehicle (b), and after J60 (c) are shown. Mean responses (n= 9; n= 6 females) for EMG AUC, peak-to-peak amplitude, tonic activity, and respiratory rate are shown in panels d-g. The DREADD ligand caused a bilateral increase in diaphragm EMG AUC, peak-to-peak amplitude, and tonic activity. For all EMG parameters, the responses were greater on the right vs. left hemi-diaphragm. Respiratory rate decreased over time. For panels d-f, the left hemi-diaphragm EMG is represented in orange, while right hemi-diaphragm EMG is blue. Error bars depict ± 1 SEM. Statistical reports for all panels are provided in Supplemental Table 2. Dia = diaphragm, AUC = area under the curve, amp = peak amplitude, BL = baseline, SL = saline (sham injection).

ChAT-Cre mice received bilateral intraspinal injections of AAV9-hSyn-DIO-hM3D(Gq)-mCherry into the ventral horns at C4. ChAT-Cre mice underwent terminal diaphragm EMG recording at the same time point and with the same protocol as wild type mice. All animals (n= 9/9) showed an increase in diaphragm EMG output in response to the J60 DREADD ligand in at least one hemi-diaphragm (Figure 2). On average, both left (**p = 0.011**; Table S2) and right (**p < 0.001**; Table S2) diaphragm EMG AUC increased over time after J60 administration (Figure 2d). Diaphragm EMG peak-to-peak amplitude had a similar, bilateral increase following J60 delivery (Left hemi-diaphragm: **p = 0.013**; Right hemi-diaphragm: **p < 0.001**; Table S2; Figure 2e). Lastly, tonic activity also showed an increase over time after J60 administration (Figure 2f) for both the left (**p= 0.002**; Table S2) and right (**p < 0.001**; Table S2) hemi-diaphragm. Respiratory rate decreased significantly over time after J60 administration (**p < 0.001**; Figure 2g; Table S2). Apart from two animals that showed a unilateral EMG response that was limited to the right hemi-diaphragm the remaining ChAT-Cre mice (n= 7/9) had bilateral increases in diaphragm EMG output following J60 administration (Figure 2).

### Wild type vs. ChAT-Cre comparison.

**Figure 3.**
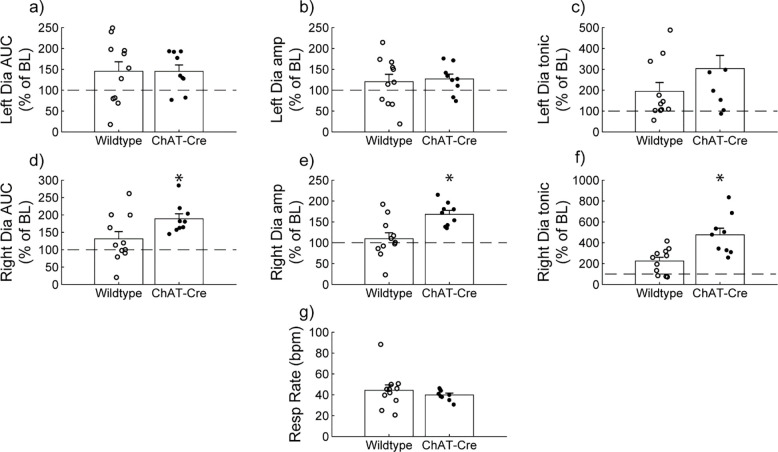
Wild type vs. ChAT-Cre mouse responses to DREADD activation. Direct comparisons of diaphragm EMG response parameters (a-f) and respiratory rate (g) at 30-minute post-J60 application (Wild type, n= 11; n= 7 females; ChAT-Cre, n= 9; n= 6 females). Left hemi-diaphragm EMG AUC (a), peak-to-peak amplitude (b) and tonic activity (c) were similar between groups. However, the same parameters on the right hemi-diaphragm (d-f) were greater in ChAT-Cre mice. Respiratory rate was similar between groups. Error bars depict ± 1 SEM. Statistical reports for all panels are provided in Supplemental Table 3. * p < 0.05. AUC = area under the curve, amp = peak EMG amplitude, Dia = diaphragm, BL = baseline, resp rate = respiratory rate.

Diaphragm EMG responses of wild type and ChAT-Cre mice were compared at the 30-minute post-J60 time point (Figure 3). Left hemi-diaphragm responses to J60 were similar between the two groups across all outcome measures (AUC: p = 0.998; Peak-to-peak amplitude: p = 0.771; Tonic activity: p = 0.160; Table S3; Figure 3a–c). However, right hemi-diaphragm responses to J60 differed across AUC (Figure 3d), peak-to-peak amplitude (Figure 3e), and tonic activity (Figure 3d) with ChAT-Cre animals on average having larger magnitude responses compared to wild type animals (AUC: **p = 0.0417**; Peak-to-peak amplitude: **p = 0.00403**; Tonic activity: **p = 0.00207**; Table S3). Respiratory rate was not different between the two groups (p = 0.382; Table S3; Figure 3g).

Five of eleven wild type mice died before the end of the experimental recording period. It is unclear if this was due to the nature of the preparation or due to an unforeseen consequence of DREADD activation. Three animals died between the 30-minute and 60-minute time points, and two animals died before the final 90-minute time point. In contrast, all mice in the ChAT-Cre cohort survived the total duration of the experimental protocol. A chi-squared assessment was run to assess the difference in survival between the two cohorts. The test did not return a p-value that met the threshold for statistical significance however, considering the small sample size the resultant p-value does indicate that there was likely some association between mouse strain (i.e., wild type, ChAT-Cre) and death after DREADD activation (Chi-squared = 3.2997, df = 1, p = 0.06929).

### ChAT-Cre rats – Plethysmography and Phrenic Nerve Recordings.

**Figure 4.**
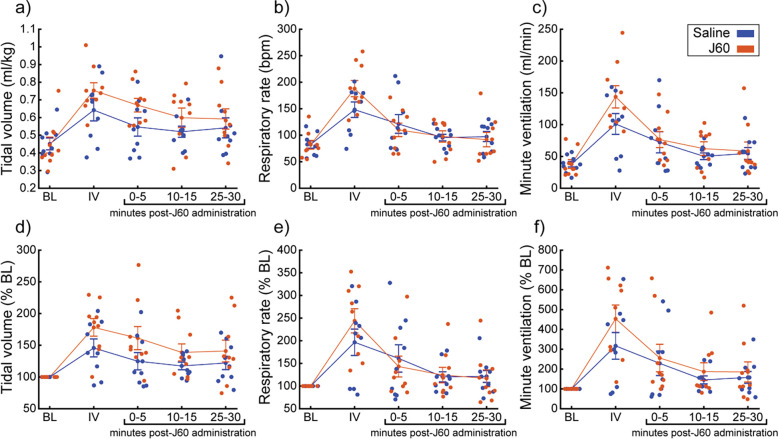
DREADD activation increases ventilation in unanesthetized ChAT-Cre rats. Summary plots (n= 9; n= 3 females) showing the impact of the J60 DREADD ligand on tidal volume, respiratory rate, and minute ventilation are shown in panels a-c. The normalized values (% of baseline) are shown in panels d-f. The DREADD ligand increased tidal volume compared to sham infusion (saline). Error bars depict ± 1 SEM. Statistical reports for all panels are provided in Supplemental Table 4. BL = baseline, IV = intravenous infusion period.

A small cohort of ChAT-Cre rats underwent diaphragm EMG recordings to ensure DREADD responses similar to mouse cohort could be obtained in rats. ChAT-Cre rats (n= 4) underwent bilateral, intraspinal injections of AAV9-hSyn-DIO-hM3D(Gq)-mCherry into the ventral horns at C4 to introduce the hM3D(Gq) DREADD transgene into phrenic motoneurons. Four of four rats showed increased diaphragm EMG output after DREADD activation (Figure S1). With that knowledge, we used a separate group of ChAT-Cre rats (n= 9; n= 3 females) to assess the effects of DREADD activation on ventilation. Whole-body plethysmography was used to measure breathing frequency, tidal volume, and minute ventilation before and after intravenous delivery of saline (sham) and J60 (Figure 4). Delivery of the J60 ligand resulted in an increase in inspiratory tidal volume compared to sham infusion (Normalized to Weight (ml/kg): Main effect of Treatment: **p= 0.037**; Figure 4a; Normalized to Baseline: Main effect of Treatment: p= 0.091; Figure 4d; Table S4). Respiratory rate appeared to be unaffected by DREADD activation and was similar between sham and J60 conditions (Respiratory Rate: Main effect of Treatment: p= 0.582; Figure 4b; Respiratory Rate Normalized to Baseline: Main effect of Treatment: p= 0.774; Figure 4e; Table S4). Minute ventilation was slightly elevated in the J60 condition vs sham; however, this increase did not reach the threshold for statistical significance (Normalized to Body Weight: Main effect of Treatment: p= 0.194; Figure 4c; Normalized to Baseline: Main effect of Treatment: p= 0.337; Figure 4f; Table S4).

Phrenic nerve recordings were made to directly assess the effects of DREADD activation on phrenic output. There was no detectable relationship between time post-AAV injection and phrenic response to DREADD activation (Pearson correlation; Left peak-to-peak response: p= 0.215; Right peak-to-peak response: p= 0.318).

**Figure 5.**
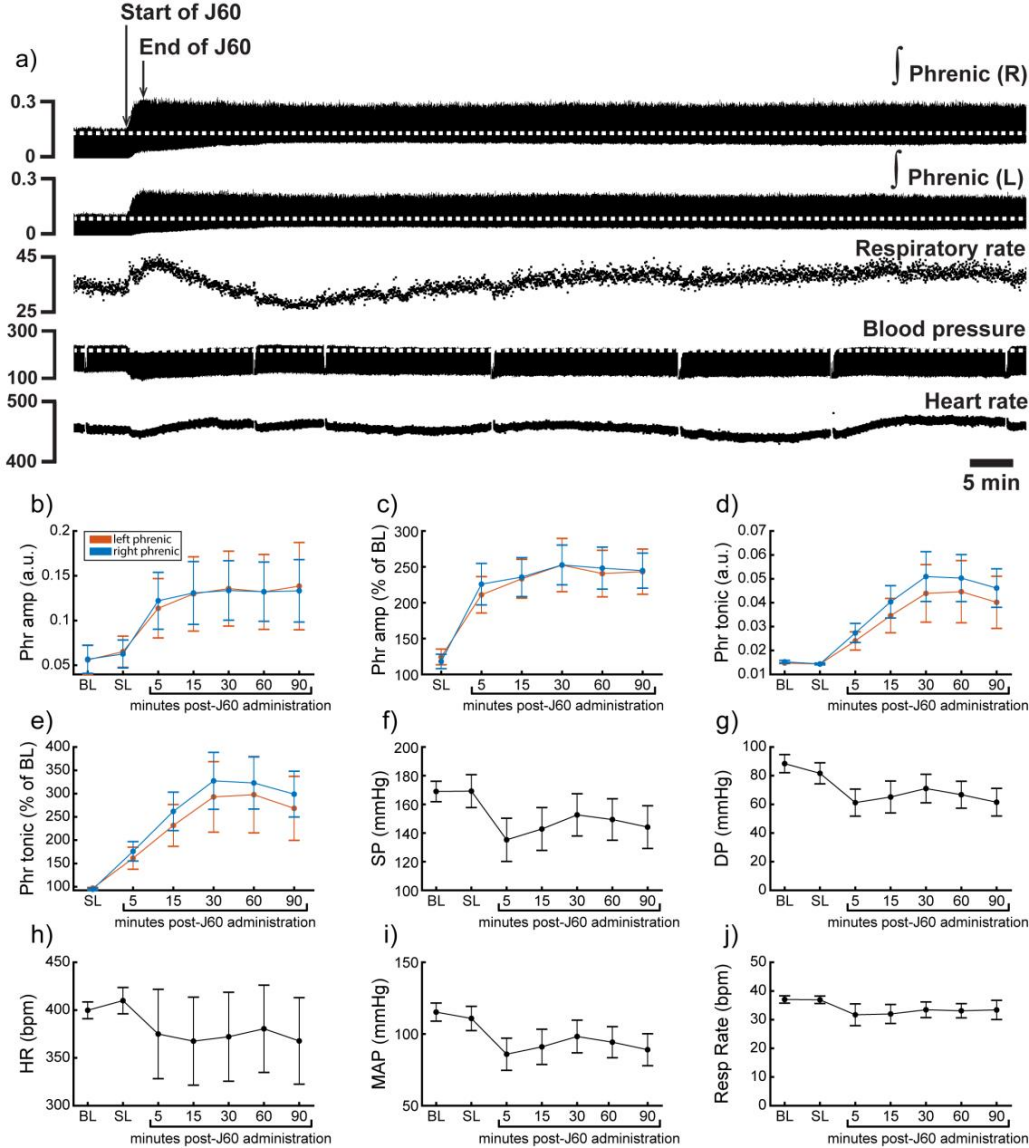
DREADD activation increases phrenic nerve output in ChAT-Cre rats. Representative data showing that the J60 DREADD ligand causes a rapid increase in phrenic nerve output (a). Mean data (n= 9; n= 3 females) showing the impact of J60 application on phrenic nerve raw (b) and normalized (c) peak-to-peak amplitude, raw (d) and normalized (e) tonic activity, systolic blood pressure (f), diastolic blood pressure (g), heart rate (h), mean arterial blood pressure (i), and respiratory rate (j). The J60 ligand caused an increase in phrenic peak-to-peak amplitude, and tonic activity. Systolic, diastolic, and mean arterial blood pressure all decreased after J60 application. Heart rate and respiratory rate were not statistically different after J60. In panels b-e, the left phrenic is represented in orange, while right phrenic is blue. Error bars depict ± 1 SEM. Statistical reports for all panels are provided in Supplemental Table 6. Phr = phrenic, amp = amplitude, BL = baseline, SP = systolic pressure, DP = diastolic pressure, HR = heart rate, MAP = mean arterial pressure.

Application of the J60 ligand caused a rapid, sustained, and bilateral increase in phrenic nerve efferent burst amplitude (Left phrenic peak-to-peak amplitude (normalized to baseline): **p< 0.001**; Right phrenic peak-to-peak amplitude (normalized to baseline): **p< 0.001**; Table S5; Figure 5b–c), whereas saline injection had no impact. The increase in phrenic burst amplitude lasted up to 100 minutes post J60 administration, at which point the experiment was terminated. Application of the J60 ligand also resulted in an increase in phrenic tonic activity (Left phrenic tonic activity (normalized to baseline): **p< 0.001**; Right phrenic tonic activity (normalized to baseline): **p< 0.001**; Table S5; Figure 5d–e).

Heart rate, systolic and diastolic blood pressure, mean arterial blood pressure (MAP), as well as respiratory rate were also assessed (Figure 5f–j). Application of J60 had no effect on heart rate (p= 0.587; Table S5; Figure 5h) or respiratory rate (p= 0.282; Table S5; Figure 5j) but did result in an decrease in both systolic (**p< 0.001**; Table S5; Figure 5f) and diastolic blood pressure (**p< 0.001**; Table S5; Figure 5g) as well as MAP (**p< 0.001**; Table S5; Figure 5i).

### Histological analysis.

**Figure 6.**
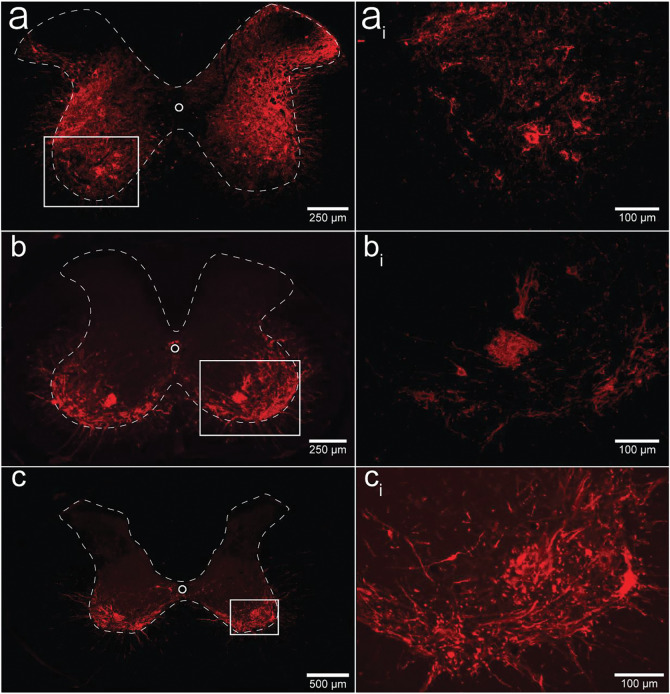
Histological assessment of mCherry expression in the C4/C5 spinal segments. Representative photomicrographs of mid-cervical spinal sections from a wild type mouse (a-a_i_), a ChAT-Cre mouse (b-b_i_) and a ChAT-Cre rat (c-c_i_). Wild type mice (a-a_i_) showed nonspecific pattern of expression throughout the mid-cervical grey matter. ChAT-Cre mice and rats (b-c_i_) showed expression limited to neurons in the ventral horns. Red color indicates positive and mCherry fluorescence. Dashed white line indicates approximate white-gray matter demarcation.

We performed qualitative analysis of the mid-cervical spinal cord from each animal to assess the extent of mCherry fluorophore expression (Figure S2). All mice from both cohorts showed evidence of mCherry expression in at least one segment of the mid-cervical spinal cord (Figure 6) except for n= 1 ChAT-Cre mouse. This mouse was excluded from analysis based off *a priori* exclusion criteria which stipulated that to be included in the final analysis an animal must show evidence of mCherry expression in the grey matter of at least one spinal segment from C3-C6. A summary of the results is given in Table 1.

Patterns of expression were relatively homogenous in wild type animals. In this cohort, the number of animals with positive mCherry expression in the grey matter increased on the rostral caudal axis. Positive mCherry counts were comparable on both the dorsal-ventral and left-right axes, with a majority of animals expression mCherry in all four quadrants. The diaphragm EMG responses to J60, on average, exhibited similarity between the left and right hemi-diaphragm in these animals, aligning with the observed pattern of mCherry expression. (Figure 1d–f).

In contrast, mCherry expression in the ChAT-Cre mice cohort was more prevalent in the ventral horns and the right side of the cord. Like the wild type animals there was a slight trend for increased mCherry expression moving rostral to caudal. These animals demonstrated a larger average DREADD response in the right hemi-diaphragm than the left (Figure 2d–f), possibly stemming from the fact that a greater number of animals exhibited mCherry expression on the right side compared to the left (Figure S3).

ChAT-Cre rats showed expression predominately in the ventral horns throughout the mid-cervical spinal cords with the highest levels of expression in spinal segments C4 and C5. One animal from the diaphragm EMG cohort showed no mCherry expression in the mid-cervical spinal cord but still had a robust increase in diaphragm output after J60 administration. All other ChAT-Cre rats showed robust mCherry expression in the ventral horns of at least one spinal segment from C3-C6. Expression in this cohort was slightly more prominent on the right side of the cord, and in the ventral horn. These histological findings were consistent with the physiology results. Although the magnitude of DREADD response between the left and right phrenic nerves for this cohort was not statistically different, there was a trend of slightly higher right phrenic tonic response compared to the left (Figure 5b–e). This trend is mirrored in the pattern of mCherry expression, where expression levels were approximately equal between spinal segments but tended to be slightly higher in the right ventral horns compared to the left.

## DISCUSSION

We describe a novel method to increase diaphragm EMG output by expressing the excitatory DREADD, hM3D(Gq), in the mid-cervical spinal cord, targeting phrenic motoneurons. Following AAV-driven expression of the DREADD in the spinal cord, application of the J60 ligand caused sustained increases in diaphragm output as measured through EMG in spontaneously breathing animals. This response was also verified using direct recordings of phrenic nerve discharge. Additionally, the DREADD ligand was able to produce an increase in inspiratory tidal volume in awake, freely behaving animals. These proof-of-concept studies provide a foundation for further development of this technology towards clinical application for restoring diaphragm activation in conditions such as cervical spinal cord injury.

### Targeted gene delivery to the phrenic motor pool.

The intraspinal AAV delivery used here was based on previous studies demonstrating successful gene delivery to phrenic motoneurons^20 24,30,31^. For example, mid-cervical spinal injections of an AAV5 vector encoding the lysosomal enzyme acid alpha-glucosidase (GAA) in animals with Pompe disease (*Gaa* null) effectively restores spinal GAA enzyme activity ^24^. Spinal-delivered viral vectors have also been used to successfully drive local expression of channelrhodopsin-2 to enable light activation of diaphragm output^20^, and to drive expression of the astrocyte glutamate transporter GLT1 in the area of the phrenic motor nuclei^30^. Other methods that have been employed to drive gene expression in phrenic motoneurons include intrapleural- and intramuscular diaphragm injection of viral vectors^32^. Intrapleural delivery requires microinjection to the “pleural space” between the visceral pleura that lines the lungs and the parietal pleura which covers the thoracic cavity. This technique^33^ effectively targets phrenic motoneurons in rodent models of cervical spinal cord injury^34–36^ and Pompe disease^37^. Intramuscular diaphragm injection allows the vector to enter phrenic nerve terminals and reach phrenic motoneuron soma via retrograde movement^32^. Direct diaphragm injection allows for a relatively high target specificity, with the gene of interest expressed almost exclusively in phrenic motoneurons (although expression can also occur in diaphragm myofibers, depending on the promoter sequence used). In pilot experiments, we tested intrapleural and also intramuscular diaphragm injections using AAV9 vectors encoding GFP (AAV9-CAG-GFP) or DREADD (AAV9-hSyn-HA-hM3D(Gq)-mCherry & AAV9-hSyn-DIO-hM3D(Gq)-mCherry). We did not, however, observe histological or physiological evidence of phrenic motoneuron transduction with these AAV9 vectors. We therefore utilized direct intraspinal injection^24,30^ to introduce the hM3D(Gq) into the phrenic motor nucleus. While this enabled proof-of-concept for targeting DREADDs to the cervical spinal cord and phrenic motoneurons, the aforementioned methods might ultimately prove better for selective phrenic motoneuron targeting. We predict that using different AAV serotypes or viruses with better retrograde movement (e.g., “AAV retro”) could optimize targeting of phrenic motoneurons^38^.

### DREADD-mediated motoneuron activation.

DREADD technology is widely used for studying brain and spinal cord neurons and networks^39,40^. Relatively few studies, however, have studied if and how DREADDs can be used to activate (or inhibit) lower motoneurons. In regard to the spinal cord, we are aware of a few prior publications^41–44^. Two of these studies used pharmacologically selective actuator module (PSAM), a type of ionotropic chemogenetic receptor, to activate thoracic^41^ and lumbar^42^ motoneurons, respectively, in mouse models of amyotrophic lateral sclerosis (ALS). In the remaining studies, excitatory DREADDs were applied to spinal motoneurons as way to improve axon regeneration follow peripheral nerve injury^43,44^. A small but growing body of work has employed DREADDs to activate hypoglossal (XII) motoneurons in the brainstem^8^. Collectively, these studies show that once hM3D(Gq) is expressed in XII motoneurons, DREADD ligands will rapidly produce an increase in the EMG activation of tongue muscles^14,15^. This increase in tongue muscle output tends to manifest as an increase in the inspiratory related activation, and tonic discharge across the respiratory cycle. Since increased tongue muscle activation can promote patency of the upper airway, XII motoneuron DREADD expression has been suggested as a possible treatment for obstructive sleep apnea^12,14,16^. For the present study, the primary innovation is the first application of DREADD technology to phrenic motoneurons. This approach was highly effective at driving sustained activation of the diaphragm muscle, and the underlying mechanisms are discussed next.

### Chemogenetic stimulation of breathing.

An important consideration is how DREADD-induced increases in the excitability of spinal neurons, including phrenic motoneurons, interacts with the endogenous neural control of breathing. Phrenic motoneurons receive a rhythmic, monosynaptic, glutamatergic synaptic input from medullary neurons. Acting via NMDA and AMPA receptors, this produces phrenic motoneuron depolarization and subsequent diaphragm muscle contraction^17^. Activating DREADD receptors on phrenic motoneurons should lower the threshold for activation via excitatory glutamatergic synaptic inputs, which would produce a greater output during the inspiratory phase. Alternatively, DREADD activation could directly lead to phrenic motoneuron action potentials even in the absence of synaptic input from the brainstem. This latter possibility could explain the tonic discharge (i.e., EMG output across the entire respiratory cycle) that was noted to occur after delivery of the DREAD ligand. Non-specific spinal cord DREADD expression, as occurred in the wild type mice (e.g., Figure 6), would likely produce an increase in the excitability and/or activation of phrenic motoneurons as well as propriospinal neurons in the immediate vicinity. Neurophysiological^45^ as well as anatomical data^23,46^ confirm synaptic connections between mid-cervical interneurons and phrenic motoneurons, making it possible that DREADD activation of these interneurons impacted the diaphragm motor response in the wild type mice.

The control of breathing is also impacted by well established “closed loop” physiologic feedback mechanisms regulating lung volume and arterial blood gases^47,48^. For example, if DREADD-induced activation of the diaphragm leads to increased alveolar ventilation, and metabolic rate is not impacted, then arterial CO_2_ values will decrease and the overall neural drive to breathe will also decrease. Vagal afferent feedback corresponding to increased lung volume also has a powerful inhibitory impact on inspiration and therefore diaphragm activation. However, the sustained increase in diaphragm EMG and tidal volume that we observed following application of the DREADD ligand indicates that these mechanisms, if activated, were not sufficient to fully inhibit the increased phrenic motoneuron output. In this regard, our additional experiments in which direct recordings of bilateral phrenic nerve discharge are informative. These nerve recording experiments were done to enable direct evaluation of the impact of spinal DREADD activation on phrenic motor output, but while keeping arterial blood gases and lung volume constant. Under these more rigorously controlled conditions, intravenous delivery of the DREADD ligand produced a rapid and sustained increase in inspiratory burst amplitude in the phrenic nerve, and with no impact on the rate of the inspiratory bursts. The relative increase in inspiratory motor output was considerably greater in the phrenic nerve recording experiments (~250% of baseline) as compared to the diaphragm EMG response in spontaneously breathing animals (~100% of baseline). This may indicate that vagal and/or blood gas related inhibitory mechanisms, as mentioned above, somewhat constrained the response to the DREADD ligand in the spontaneously breathing animal.

### Critique of methods.

There are a few caveats that should be discussed. First, the precision of the AAV delivery could be improved by further refining spinal injection surgical techniques. In the current study we used a stereotaxic frame and previously validated coordinates^24,25^ to guide the intraspinal AAV injections. However, we observed variability in the (left vs. right) laterality of mid-cervical mCherry expression as well as the physiological response to the DREADD ligand, particularly in the ChAT-Cre mice (e.g., Figure 2; Table 1). This could have occurred due to subtle variations of the positioning of the animal within the stereotaxic frame, and/or placement of the needle tip, leading to slight deviations for the desired coordinates between the left and right phrenic nuclei. Second, we did not unequivocally verify that the DREADD was expressed in phrenic motoneurons using retrograde labeling methods^33,49^. However, the phrenic motor nucleus has been well described in the mouse^50^ and the rat^51,52^, and the fluorophore (mCherry) expression observed in our experiments is very clearly in the expected location of phrenic motoneurons (Figure 6b–c_i_; Supplemental Figure 2). Further, the robust increase in phrenic motor output after the DREADD ligand, particularly in the ChAT-Cre rat experiment (Figure 5) is further evidence of effective phrenic motoneuron targeting.

### Conclusion.

Collectively, the data support the conclusion that cervical spinal cord directed chemogenetic methods can be used to produce a sustained increase in phrenic motor output, diaphragm activation, and inspiratory tidal volume. However, more direct targeting of DREADD expression to phrenic motoneurons may likely to be needed for optimal diaphragm activation using this method. In this regard, improvement of the AAV delivery methods will increase the selectively of the approach for more precise targeting of phrenic motoneurons. In regard to the “translational value” of this work, spinal cord chemogenetics may have application to clinical conditions associated with an inability to activate the diaphragm. For example, incomplete cervical spinal cord injury is a condition in which the bulbospinal synaptic inputs to phrenic motoneurons are interrupted. After cervical spinal cord injury, expressing an excitatory DREADD in phrenic motoneuron could be used to increase the excitability of these cells, and thereby improve the efficacy of the limited bulbospinal synaptic inputs.

## Supplementary Material

Supplement 1

## Figures and Tables

**Table 1.** Qualitative assessment of mCherry expression in the mid-cervical spinal cord. Spinal segments C3-C6 were assessed in quadrants broken into dorsal, ventral, left, and right. Spinal segments were counted as “positive” if they showed any evidence of mCherry expression in neuronal soma or fibers. The counts therefore indicate the number of animals of a given cohort that were mCherry positive for a given spinal segment quadrant. All animals showed slight trend for more mCherry expression moving rostral to caudal and for more expression in the ventral vs the dorsal lamina. This trend was more prominent in the ChAT-Cre animals. At the bottom of the table, a heatmap is provided for easier assessment of the distribution of positive mCherry counts across quadrants and spinal segments.

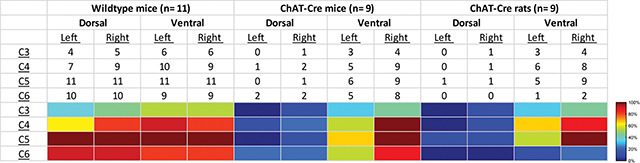

## Data Availability

The datasets along with MATLAB and R code generated during the current study are available from the corresponding author upon reasonable request.

## Abbreviations:

DREADD: designer receptors exclusively activated by designer drugs
EMG: electromyography
AUC: area under the curve
J60: JHU37160
AAV: adeno-associated virus
VT: tidal volume
ChAT: choline acetyltransferase
Cre: Cre recombinase
