# Supplementary material for: Chemogenetic stimulation of phrenic motor output and diaphragm activity
Source: bioRxiv. 2024 Apr 15:2024.04.12.589188. Preprint. [Version 1] doi: 10.1101/2024.04.12.589188 (PMC11042184; doi:10.1101/2024.04.12.589188)
Supplement: Supplementary file 1 [file NIHPP2024.04.12.589188v1-supplement-1.pdf]

bioRxiv preprint doi: https://doi.org/10.1101/2024.04.12.589188; this version posted April 15, 2024. The copyright holder for this preprint (which was not certified by peer review) is the author/funder, who has granted bioRxiv a license to display the preprint in perpetuity. It is made available under aCC-BY 4.0 International license.

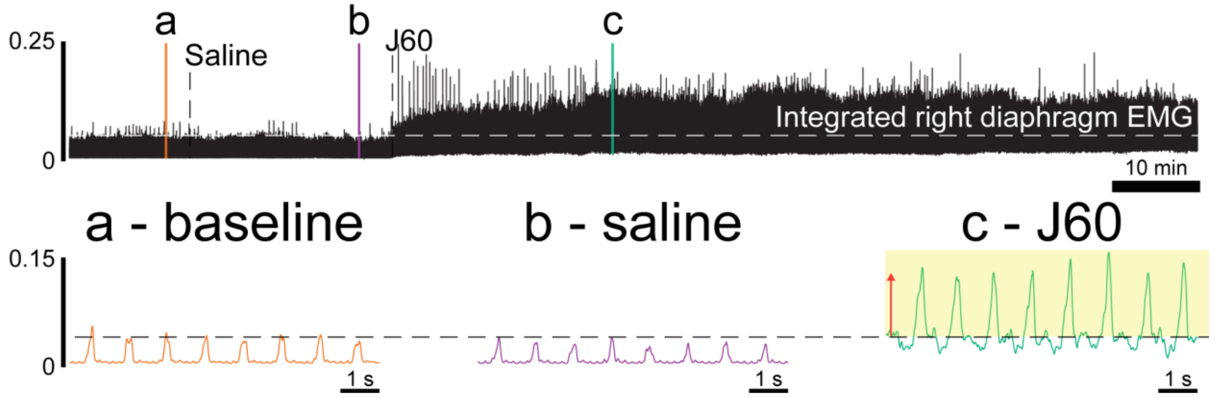

Supplemental Figure 1. ***Impact of DREADD activation on diaphragm EMG in ChAT-Cre rats.*** Example trace of rectified and integrated diaphragm EMG activity from a ChAT-Cre rat that had previously undergone injections of AAV9-hSyn-DIO-hM3D(Gq)-mCherry into the C4 ventral horns before and after application of the DREADD ligand, J60. Callout panels below show example activity at baseline (a), after injection of vehicle (b), and after application of the DREADD ligand, J60 (c).

bioRxiv preprint doi: https://doi.org/10.1101/2024.04.12.589188; this version posted April 15, 2024. The copyright holder for this preprint (which was not certified by peer review) is the author/funder, who has granted bioRxiv a license to display the preprint in perpetuity. It is made available under aCC-BY 4.0 International license.

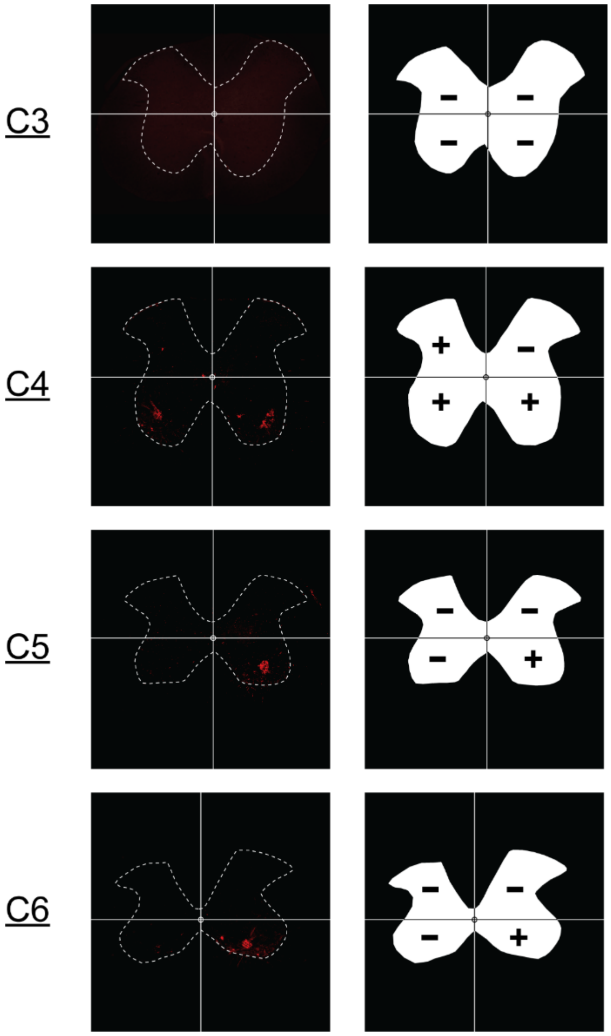

Supplemental Figure 2. ***Example quantification of mCherry expression in the C3-C6 spinal cord.*** Mid-cervical spinal cord images from ChAT-Cre animal (left) with positive mCherry expression. A grid has been placed on each image to highlight the quadrant system (i.e., left dorsal, right dorsal, left ventral, right ventral) used to score the tissue. The right column shows an outline of each section on the left with a score for each quadrant based on mCherry expression. A given quadrant was scored with a "+" if it contained any mCherry positive neurons or fibers else it was given a "-" signifying no mCherry expression.

bioRxiv preprint doi: https://doi.org/10.1101/2024.04.12.589188; this version posted April 15, 2024. The copyright holder for this preprint (which was not certified by peer review) is the author/funder, who has granted bioRxiv a license to display the preprint in perpetuity. It is made available under aCC-BY 4.0 International license.

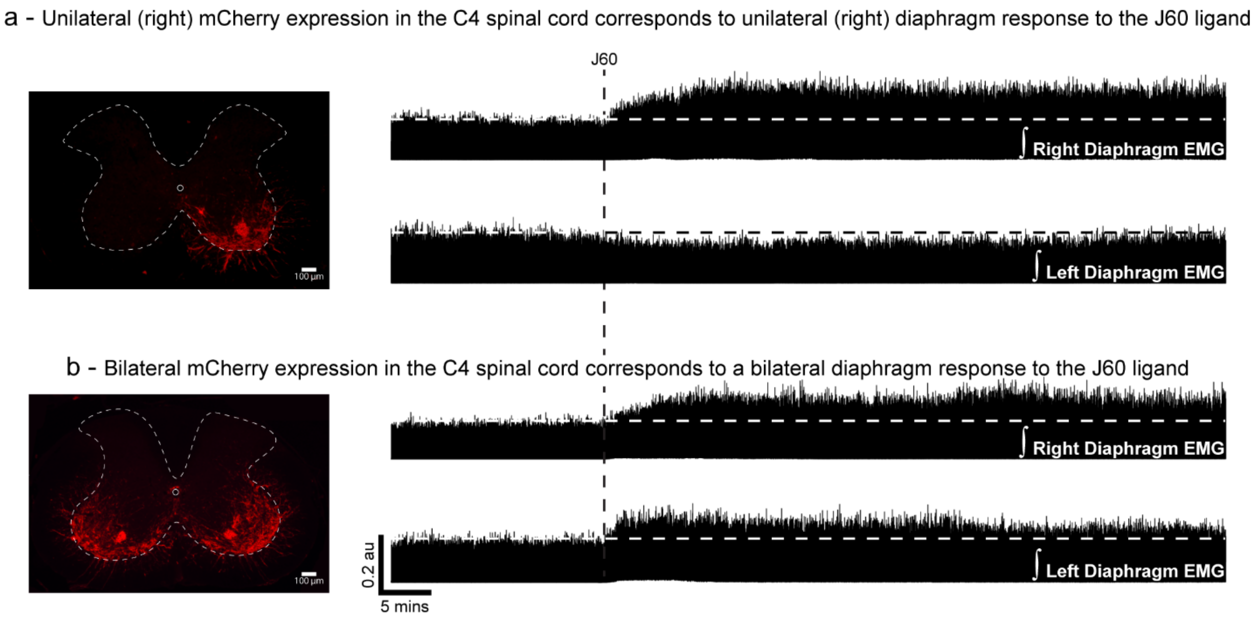

Supplemental Figure 3. ***mCherry expression in the mid-cervical ventral horn corresponds with the laterality of the diaphragm EMG DREADD response*****.** Example spinal histology (left) from two ChAT-Cre mice showing unilateral (a) and bilateral (b) expression of mCherry (red) in the ventral horn(s) and corresponding diaphragm EMG traces. Unilateral ventral horn mCherry expression (a) results in DREADD responses that were limited to the ipsilateral hemi-diaphragm. Animals with bilateral ventral horn DREADD expression responded to DREADD activation with a bilateral increase in diaphragm output.

bioRxiv preprint doi: https://doi.org/10.1101/2024.04.12.589188; this version posted April 15, 2024. The copyright holder for this preprint (which was not certified by peer review) is the author/funder, who has granted bioRxiv a license to display the preprint in perpetuity. It is made available under aCC-BY 4.0 International license.

Main Effects

Outcome	Hemi-diaphragm	Test	df	Test statistic	p value
Diaphragm EMG AUC	left	One-way RM ANOVA	6,68	F = 4.008	**0.002**
	right	One-way RM ANOVA	6,68	F = 4.054	**0.002**
Diaphragm EMG peak-to-peak amplitude	left	One-way RM ANOVA	6,68	F = 2.213	0.056
	right	One-way RM ANOVA	6,68	F = 3.191	**0.01**
Diaphragm EMG tonic activity	left	One-way RM ANOVA	6,68	F = 2.256	0.052
	right	One-way RM ANOVA	6,68	F = 5.225	**< 0.001**
Respiratory Rate	NA	One-way RM ANOVA	6,68	F = 0.419	0.863

Post-Hoc Tests (Tukey Test)

Outcome	Hemi-diaphragm	Comparison	Diff of Ranks	q	P
Diaphragm EMG AUC	left	15 min vs. Saline	60.488	4.972	**0.015**
		60 min vs. Saline	68.183	5.064	**0.013**
		90 min vs. Saline	66.079	4.446	**0.041**
Diaphragm EMG AUC	right	15 min vs. Saline	41.481	4.41	**0.044**
		60 min vs. Saline	54.104	5.197	**0.01**
		90 min vs. Saline	51.214	4.457	**0.04**
Diaphragm EMG peak-to-peak amplitude	right	90 min vs. Saline	40.925	4.947	**0.016**
Diaphragm EMG tonic activity	right	15 min vs. Baseline	124.737	5.044	**0.013**
		15 min vs. Saline	135.783	5.49	**0.005**
		30 min vs. Baseline	110.583	4.471	**0.039**
		30 min vs. Saline	121.628	4.918	**0.017**
		90 min vs. Baseline	140.208	4.641	**0.029**
		90 min vs. Saline	151.254	5.006	**0.014**

Supplemental Table 1. ***Statistical summary for impact of DREADD activation on diaphragm EMG in wild type mice.*** Time points are in reference to minutes passed since J60 infusion. Summary data presented in Figure 1. EMG = electromyography, AUC = area under the curve, RM = repeated measures, df = degrees of freedom. Bolded p-values indicates p<0.05.

bioRxiv preprint doi: https://doi.org/10.1101/2024.04.12.589188; this version posted April 15, 2024. The copyright holder for this preprint (which was not certified by peer review) is the author/funder, who has granted bioRxiv a license to display the preprint in perpetuity. It is made available under aCC-BY 4.0 International license.

Main Effects

Outcome	Hemi-diaphragm	Test	df	Test statistic	p value
Diaphragm EMG AUC	left	Friedman RM analysis on ranks	6	Chi-square = 16.619	**0.011**
	right	Friedman RM analysis on ranks	6	Chi-square = 40.143	**< 0.001**
Diaphragm EMG peak-to-peak amplitude	left	One-way RM ANOVA	6,62	F = 3.05	**0.013**
	right	Friedman RM analysis on ranks	6	Chi-square = 36.667	**< 0.001**
Diaphragm EMG tonic activity	left	Friedman RM analysis on ranks	6	Chi-square = 20.952	**0.002**
	right	One-way RM ANOVA	6,62	F = 10.076	**< 0.001**
Respiratory Rate	NA	One-way RM ANOVA	6,62	F = 25.13	**< 0.001**

Post-Hoc Tests (Tukey Test)

Outcome	Hemi-diaphragm	Comparison	Diff of Ranks	q	P
Diaphragm EMG AUC	left	30 min vs. Saline	32	4.938	**0.009**
Diaphragm EMG AUC	right	15 min vs. Baseline	31	4.783	**0.013**
		15 min vs. Saline	38	5.864	**<0.001**
		30 min vs. Baseline	35	5.401	**0.003**
		30 min vs. Saline	42	6.481	**<0.001**
		60 min vs. Baseline	32	4.938	**0.009**
		60 min vs. Saline	39	6.018	**<0.001**
		90 min vs. Baseline	27	4.166	**0.05**
		90 min vs. Saline	34	5.246	**0.004**
Diaphragm EMG peak-to-peak amplitude	left	30 min vs. Saline	32.824	4.447	**0.042**
Diaphragm EMG peak-to-peak amplitude	right	15 min vs. Baseline	31	4.783	**0.013**
		15 min vs. Saline	36	5.555	**0.002**
		30 min vs. Baseline	34	5.246	**0.004**
		30 min vs. Saline	39	6.018	**<0.001**
		60 min vs. Baseline	32	4.938	**0.009**
		60 min vs. Saline	37	5.709	**0.001**
		90 min vs. Saline	31	4.783	**0.013**
Diaphragm EMG tonic activity	left	5 min vs. Baseline	28	4.32	**0.037**
		15 min vs. Baseline	29	4.475	**0.026**
		90 min vs. Baseline	35	5.401	**0.003**
Diaphragm EMG tonic activity	right	5 min vs. Baseline	295.193	5.582	**0.005**
		5 min vs. Saline	297.174	5.619	**0.004**
		15 min vs. Baseline	375.713	7.104	**<0.001**
		15 min vs. Saline	377.694	7.142	**<0.001**
		30 min vs. Baseline	347.212	6.565	**<0.001**
		30 min vs. Saline	349.193	6.603	**<0.001**
		60 min vs. Baseline	317.232	5.998	**0.002**
		60 min vs. Saline	319.213	6.036	**0.002**
		90 min vs. Baseline	358.443	6.778	**<0.001**
		90 min vs. Saline	360.424	6.815	**<0.001**
Respiratory Rate	NA	5 min vs. 30 min	5.296	4.733	**0.025**
		5 min vs. 60 min	7.296	6.52	**<0.001**
		5 min vs. 90 min	11.889	10.624	**<0.001**
		15 min vs. Baseline	5.778	5.163	**0.011**
		15 min vs. Saline	5.963	5.328	**0.008**
		15 min vs. 90 min	9.222	8.241	**<0.001**
		30 min vs. Baseline	8.407	7.513	**<0.001**
		30 min vs. Saline	8.593	7.678	**<0.001**
		30 min vs. 90 min	6.593	5.891	**0.002**
		60 min vs. Baseline	10.407	9.3	**<0.001**
		60 min vs. Saline	10.593	9.465	**<0.001**
		90 min vs. Baseline	15	13.404	**<0.001**
		90 min vs. Saline	15.185	13.569	**<0.001**

Supplemental Table 2. ***Statistical summary for impact of DREADD activation on diaphragm EMG in ChAT-Cre mice.*** Time points are in reference to minutes passed since J60 infusion. Summary data presented in Figure 2. EMG = electromyography, AUC = area under the curve, RM = repeated measures, df = degrees of freedom. Bolded p-values indicates p<0.05.

bioRxiv preprint doi: https://doi.org/10.1101/2024.04.12.589188; this version posted April 15, 2024. The copyright holder for this preprint (which was not certified by peer review) is the author/funder, who has granted bioRxiv a license to display the preprint in perpetuity. It is made available under aCC-BY 4.0 International license.

Outcome	Hemi-diaphragm	Test	df	Test statistic	p value
Diaphragm EMG AUC	left	Student's t-test	18	t = 0.00310	p = 0.998
	right	Student's t-test	18	t = -2.913	**p = 0.0417**
Diaphragm EMG peak-to-peak amplitude	left	Student's t-test	18	t = -0.295	p = 0.771
	right	Student's t-test	18	t = -3.294	**p = 0.00403**
Diaphragm EMG tonic activity	left	Mann-Whitney Rank Sum test	18	Mann-Whitney U Statistic= 31.000	p = 0.160
	right	Student's t-test	18	t = -3.596	**p = 0.00207**
Respiratory Rate	NA	Mann-Whitney Rank Sum test	18	Mann-Whitney U Statistic= 38.000	p = 0.382

Supplemental Table 3. ***Statistical summary for impact of DREADD activation on diaphragm EMG in in wild type mice vs. ChAT-Cre mice at the 30-min post J60 infusion time point.*** Summary data presented in Figure 3. EMG = electromyography, AUC = area under the curve, df = degrees of freedom. Bolded p-values indicates $p<0.05$.

bioRxiv preprint doi: https://doi.org/10.1101/2024.04.12.589188; this version posted April 15, 2024. The copyright holder for this preprint (which was not certified by peer review) is the author/funder, who has granted bioRxiv a license to display the preprint in perpetuity. It is made available under aCC-BY 4.0 International license.

Outcome	Test	Normalization	Main effects	df	Test statistic	p value
Tidal Volume	Two-way RM ANOVA	Normalized to body weight (ml/kg)	Treatment	1,89	F = 6.286	**p= 0.037**
			Time	4,89	F = 9.817	**p < 0.001**
			Interaction	4,89	F = 0.969	p= 0.438
		Normalized to baseline	Treatment	1,89	F = 3.7	p= 0.091
			Time	4,89	F = 9.448	**p< 0.001**
			Interaction	4,89	F = 1.049	p= 0.398
Respiratory Rate	Two-way RM ANOVA	Raw values	Treatment	1,89	F = 0.329	p= 0.582
			Time	4,89	F = 14.326	**p < 0.001**
			Interaction	4,89	F = 1.640	p= 0.188
		Normalized to baseline	Treatment	1,89	F = 0.0885	p= 0.774
			Time	4,89	F = 11.862	**p< 0.001**
			Interaction	4,89	F = 1.310	p= 0.287
Minute Ventilation	Two-way RM ANOVA	Normalized to body weight (ml/kg)	Treatment	1,89	F = 2.011	p= 0.194
			Time	4,89	F = 12.875	**p < 0.001**
			Interaction	4,89	F = 1.215	p= 0.324
		Normalized to baseline	Treatment	1,89	F = 1.043	p= 0.337
			Time	4,89	F = 10.776	**p< 0.001**
			Interaction	4,89	F = 0.490	p= 0.490

Supplemental Table 4. ***Statistical summary for impact of DREADD activation on plethysmography outcomes in unanesthetized ChAT-Cre rats using two-way repeated measures ANOVAs.*** Each outcome measure is presented normalized to body weight (with the exception of respiratory rate) and normalized to values at baseline. Summary data presented in Figure 4. RM = repeated measures, ml/kg = milliliters of air per kilogram of animal's body weight, df = degrees of freedom. Bolded p-values indicates $p<0.05$.

bioRxiv preprint doi: https://doi.org/10.1101/2024.04.12.589188; this version posted April 15, 2024. The copyright holder for this preprint (which was not certified by peer review) is the author/funder, who has granted bioRxiv a license to display the preprint in perpetuity. It is made available under aCC-BY 4.0 International license.

Main Effects

Outcome	Nerve	Test	df	Test statistic	p value
Phrenic peak-to-peak amplitude (normalized to baseline)	left	One-way RM ANOVA	6,62	F = 13.355	**p< 0.001**
	right	Friedman Repeated Measures Analysis of Variance on Ranks	6	Chi-square = 35.619	**p< 0.001**
Phrenic tonic activity (normalized to baseline)	left	Friedman Repeated Measures Analysis of Variance on Ranks	6	Chi-square = 36.714	**p< 0.001**
	right	One-way RM ANOVA	6,62	F = 14.544	**p< 0.001**
Heart rate	NA	Friedman Repeated Measures Analysis of Variance on Ranks	6	Chi-square = 4.667	p= 0.587
Systolic blood pressure	NA	One-way RM ANOVA	6,62	F = 5.403	**p< 0.001**
Diastolic blood pressure	NA	One-way RM ANOVA	6,62	F = 12.460	**p< 0.001**
Mean arterial blood pressure	NA	One-way RM ANOVA	6,62	F = 9.422	**p< 0.001**
Respiratory Rate	NA	One-way RM ANOVA	6,62	F = 1.285	p= 0.282

Post-Hoc Tests (Tukey Test)

Outcome	Nerve	Comparison	Diff of Ranks	q	P
Phrenic peak-to-peak amplitude (normalized to baseline)	left	5 min vs. Baseline	111.026	6.52	**<0.001**
		5 min vs. Saline	86.692	5.091	**0.013**
		15 min vs. Baseline	133.337	7.83	**<0.001**
		15 min vs. Saline	109.003	6.401	**<0.001**
		30 min vs. Baseline	152.488	8.955	**<0.001**
		30 min vs. Saline	128.153	7.526	**<0.001**
		60 min vs. Baseline	140.658	8.26	**<0.001**
		60 min vs. Saline	116.324	6.831	**<0.001**
		90 min vs. Baseline	143.291	8.415	**<0.001**
		90 min vs. Saline	118.956	6.986	**<0.001**
	right	15 min vs. Baseline	32	4.938	**0.009**
		15 min vs. Saline	29	4.475	**0.026**
		30 min vs. Baseline	37	5.709	**0.001**
		30 min vs. Saline	34	5.246	**0.004**
		60 min vs. Baseline	35	5.401	**0.003**
		60 min vs. Saline	32	4.938	**0.009**
		90 min vs. Baseline	35	5.401	**0.003**
		90 min vs. Saline	32	4.938	**0.009**
Phrenic tonic activity (normalized to baseline)	left	30 min vs. Baseline	32	4.938	**0.009**
		30 min vs. Saline	42	6.481	**<0.001**
		60 min vs. Baseline	33	5.092	**0.006**
		60 min vs. Saline	43	6.635	**<0.001**
		90 min vs. Saline	31	4.783	**0.013**
	right	15 min vs. Baseline	161.81	6.077	**0.002**
		15 min vs. Saline	167.132	6.277	**0.001**
		30 min vs. Baseline	227.376	8.54	**<0.001**
		30 min vs. Saline	232.698	8.74	**<0.001**
		30 min vs. 5 min	151.316	5.683	**0.004**
		60 min vs. Baseline	222.864	8.37	**<0.001**
		60 min vs. Saline	228.186	8.57	**<0.001**
		60 min vs. 5 min	146.805	5.514	**0.005**
		90 min vs. Baseline	198.832	7.468	**<0.001**
		90 min vs. Saline	204.154	7.668	**<0.001**
		90 min vs. 5 min	122.773	4.611	**0.031**
Systolic blood pressure	NA	5 min vs. Baseline	33.737	6.017	**0.002**
		5 min vs. Saline	33.977	6.06	**0.002**
		15 min vs. Baseline	26.184	4.67	**0.028**
		15 min vs. Saline	26.423	4.713	**0.026**
		90 min vs. Baseline	24.843	4.431	**0.043**
		90 min vs. Saline	25.083	4.474	**0.04**
Diastolic blood pressure	NA	5 min vs. Baseline	27.201	9.186	**<0.001**
		5 min vs. Saline	20.438	6.902	**<0.001**
		15 min vs. Baseline	23.277	7.861	**<0.001**
		15 min vs. Saline	16.515	5.577	**0.005**
		30 min vs. Baseline	17.364	5.864	**0.003**
		60 min vs. Baseline	21.693	7.326	**<0.001**
		60 min vs. Saline	14.931	5.042	**0.014**
		90 min vs. Baseline	26.929	9.094	**<0.001**
		90 min vs. Saline	20.167	6.811	**<0.001**
Mean arterial blood pressure	NA	5 min vs. Baseline	29.379	8.053	**<0.001**
		5 min vs. Saline	24.951	6.839	**<0.001**
		15 min vs. Baseline	24.246	6.646	**<0.001**
		15 min vs. Saline	19.818	5.432	**0.006**
		30 min vs. Baseline	17.025	4.667	**0.028**
		60 min vs. Baseline	20.993	5.754	**0.003**
		60 min vs. Saline	16.565	4.54	**0.036**
		90 min vs. Baseline	26.234	7.191	**<0.001**
		90 min vs. Saline	21.806	5.977	**0.002**

Supplemental Table 5. ***Statistical summary for impact of DREADD activation on phrenic nerve activity in ChAT-Cre mice.*** Time points are in reference to minutes passed since J60 infusion. Summary data presented in Figure 5. RM = repeated measures, df = degrees of freedom. Bolded p-values indicates p<0.05.
